# Ketoprofen‐induced photoallergic consort contact dermatitis: A difficult diagnosis

**DOI:** 10.1111/cod.14058

**Published:** 2022-02-13

**Authors:** Astrid Herzum, Emanuele Cozzani, Aurora Parodi, Rosella Gallo

**Affiliations:** ^1^ Division of Dermatology, Department of Health Sciences (DISSAL) University of Genoa, Genoa Italy, Ospedale Policlinico San Martino IRCCS Genoa Italy

**Keywords:** allergic contact dermatitis, case report, consort dermatitis, ketoprofen, patch test, photo‐contact dermatitis

## CASE REPORT

Consort contact dermatitis is an allergic or photoallergic dermatitis caused by sensitizers conveyed by a partner or companion via direct interpersonal skin contact or indirect contact through shared objects.^1^ We describe a patient who experienced severe bouts of photoallergic contact dermatitis as a result of an indirect contact with topical ketoprofen used by her mother.

A 34‐year‐old lady was referred with a 7‐year history of vesico‐bullous eruptions recurrent on different body sites, presently in remission.

**FIGURE 1:**
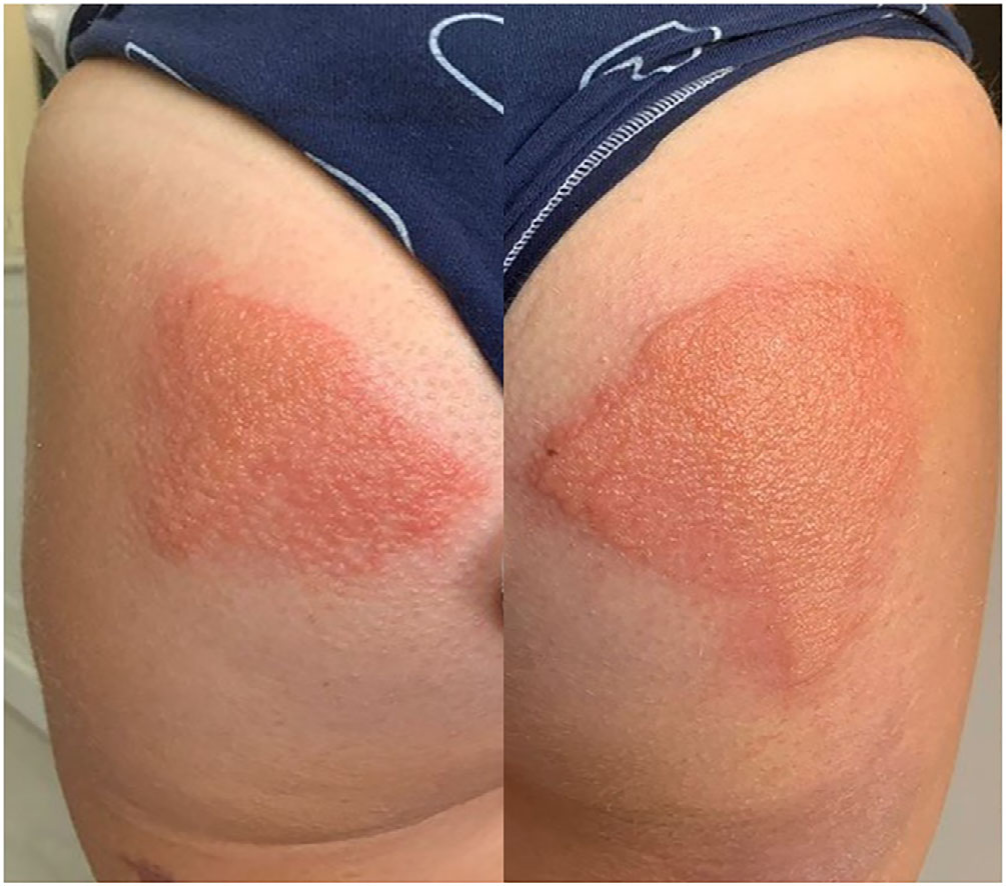
Well‐demarcated erythemato‐vesico‐bullous lesions that had developed on the patient’s buttocks during a seaside holiday where she sometimes sat on the same lounge chair used by her mother

She showed photos of her latest eruption documenting well‐circumscribed erythemato‐vesico‐bullous lesions on her buttocks (Figure 1). These had occurred during a seaside holiday.

Throughout the years she had undergone several dermatological consultations and investigations, including patch testing, histopathology, and direct immunofluorescence of lesional skin. All were inconclusive but for a histological picture of spongiosis and weak patch test reactions to nickel and fragrance mix without any current relevance.

The history was suggestive of recurrent acute allergic contact dermatitis induced by a strong allergen but the patient denied direct contact with any likely trigger including cosmetics and topical medicaments.

A meticulous search for indirect contact with renowned potent haptens eventually revealed that the patient’s mother used topical ketoprofen and accompanied her daughter to the beach where they happened to use the same lounge chair.

Upon specific enquiry, the patient remembered that 20 years before acute eczema had occurred on one of her knees after the application of a ketoprofen‐containing cream.

Photo‐patch testing was performed in accordance with the Italian Guidelines in Patch Testing.^2^ In particular, we applied a photo‐patch series that contained several sunscreen agents including benzophenone‐3, benzophenone‐4, and octocrylene, all 10% pet. The allergens were applied in duplicate on the upper back; one set was irradiated on day (D) 2 with UVA radiation at the dose of 5 J/cm^2^. Readings were taken on D2, D4, and D7. The patient reacted to ketoprofen 2.5% pet. (+++) on the irradiated site. No other positive reaction was observed. The patient’s dermatitis never recurred after her mother discontinued topical ketoprofen.

## DISCUSSION

Ketoprofen, a nonsteroidal anti‐inflammatory drug, is a renowned photo‐contact allergen,^3^, ^4^ possibly cross‐reacting with cinnamyl alcohol and co‐reacting with octocrylene or benzophenones.^5^ In our patient no concomitant photoallergic contact dermatitis from sunscreens was present. Consort photo‐allergic contact dermatitis from ketoprofen is rarely reported^6^, ^7^, ^8^ and is possibly underdiagnosed. Our case shows that it can remain undetected for years unless it is specifically suspected and investigated.

## CONSENT FOR PUBLICATION

Written informed consent was obtained from the patient for publication of this case report and any accompanying images.

## AUTHOR CONTRIBUTIONS

**Astrid Herzum:** Conceptualization (equal); data curation (equal); formal analysis (equal); investigation (equal); methodology (equal); project administration (equal); supervision (equal); validation (equal); visualization (equal); writing – original draft (equal); writing – review and editing (equal). **Emanuele Cozzani:** Conceptualization (equal); data curation (equal); supervision (equal); validation (equal). **Aurora Parodi:** Conceptualization (equal); data curation (equal); resources (equal); supervision (equal); validation (equal); writing – review and editing (equal). **Rosella Gallo:** Conceptualization (equal); data curation (equal); formal analysis (equal); investigation (equal); methodology (equal); project administration (equal); supervision (equal); validation (equal); visualization (equal); writing – original draft (equal); writing – review and editing (equal).

## CONFLICTS OF INTEREST

The authors declare no conflict of interest.
